# A suture technique for easier reduction and repair of bucket-handle meniscal tears while using the all-inside devices

**DOI:** 10.1051/sicotj/2016035

**Published:** 2016-11-29

**Authors:** Engin Çetinkaya, Ersin Kuyucu, Murat Gül, Osman Lapçin, Kutalmış Albayrak, Sarper Gürsu

**Affiliations:** 1 Baltalimani Bone and Joint Diseases Training and Research Hospital, Department of Ortopaedics and Traumatology 34470 Istanbul Turkey; 2 Department of Orthopaedics and Traumatology, Medipol University 34214 Istanbul Turkey

**Keywords:** Bucked-handle, Meniscal tear, All-inside, Inside-out, Meniscal repair

## Abstract

Arthroscopic repair of bucket-handle meniscal tears is difficult due to their complex pathology. Many meniscal repair techniques such as all-inside, inside-out, and outside-in have been described for the treatment of these tears. Loss of reduction is a likely complication with the use of new-generation, all-inside suture instruments, as the tip of the needle is extracted following advancement of the first implant behind the capsule. The complication may be encountered quite often and renders the use of the meniscus repair instrument unusable and causes an irreparable iatrogenic injury in the meniscus. The application of a simpler and more efficient technique is necessary until surgical experience is gained. The aim of this study was to define a new, simpler, and more efficient combination of suturing method in the treatment of bucket-handle meniscal repairs and minimize the rate of complications which may be caused by this technique.

## Introduction

Bucket-handle meniscal tears comprise about 10% of all tears [1]. Arthroscopic repair of the bucket-handle meniscal tears is difficult due to their complex pathology. Many meniscal repair techniques such as all-inside, inside-out, and outside-in have been described for the treatment of these tears [2–6]. At present, it can be concluded that no single meniscal repair technique or instrument is superior in all situations [7]. On the other hand, with the developing technology, the decreased complication rate of all-inside meniscal repair instruments led to a greater utilization of these repair techniques.

**Figure 1.**
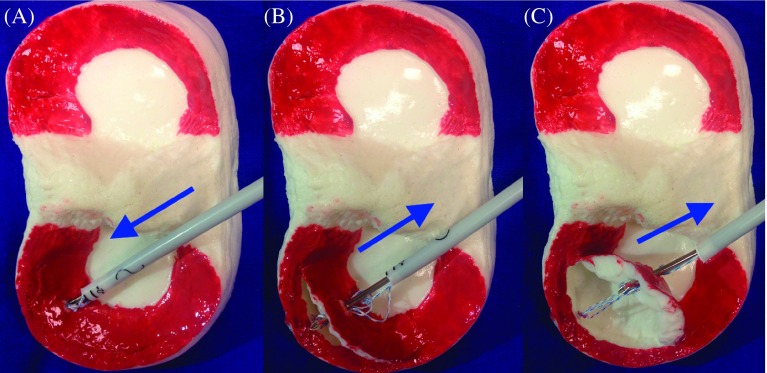
Schematic illustration of the repair of bucket-handle tears with the all-inside method. (A) Insertion of the first suture. (B) Fixation of the first suture on the meniscus as the hand instrument is being removed. (C) The hand instrument causing failure of reduction.

Loss of reduction is a likely complication with the use of new-generation, all-inside suture instruments, as the tip of the needle is extracted following advancement of the first implant behind the capsule (Figure 1). The complication may be encountered quite often and renders the use of the meniscus repair instrument unusable and causes an irreparable iatrogenic injury in the meniscus. The application of a simpler and more efficient technique is necessary until surgical experience is gained.

The aim of this study was to define a new, simpler, and more efficient combination of suturing method in the treatment of bucket-handle meniscal repairs and minimize the rate of complications which may be caused by this technique.

## Surgical technique

The patient was placed in the supine position and a tourniquet was applied on the appropriate lower extremity. The knees of the patients were positioned in 90° of flexion and fixed with fixators. Standard knee arthroscopy equipment was employed for routine diagnostic arthroscopy primarily, using anterolateral and anteromedial portals.

**Figure 2.**
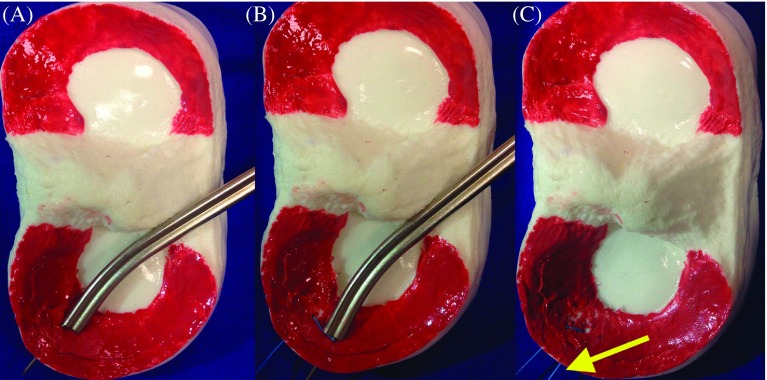
Application of the inside-out suture as the first suture in our hybrid technique. (A) Insertion of the first leg of the suture from the posterior. (B) Insertion of the second leg of the suture from the anterior. (C) Fixation of the suture with the meniscus reduced.

**Figure 3.**
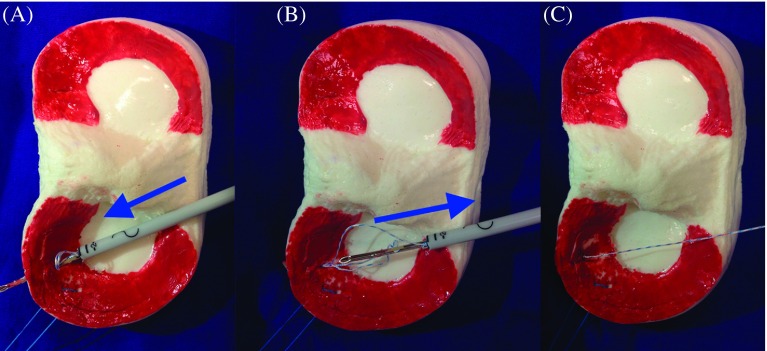
Application of the second all-inside suture in our hybrid technique. (A) Insertion of the suture. (B) The unfixed suture will easily come off as the hand instrument is being removed. (C) The reduced meniscus can easily be fixed.

The camera was inserted through the anterolateral portal and hand instruments through the anteromedial portal for the bucket-handle tear of the meniscus. First, the edges of the tears were debrided with a motorized shaver and rasp. After reduction was achieved with the inspection probe, the camera was inserted through the anteromedial portal, and the inside-out suture cannula, inserted through the anterolateral portal, was aligned with the intersection point of the posterior meniscus and its corpus. The first needle was inserted through the cannula from the anterior direction and close to the capsule and the second from the posterior direction and then it was removed (Video Supplement) (Ticron 2-0, Covidien, Dublin, Ireland). A longitudinal skin incision of 2 cm was made over the point where the needles were removed, the dissection was extended to the capsule in a fashion to protect the neurovascular structures, and the sutures were tied and fixed on the capsule. With the fixation of inside-out sutures on the capsule, the displacement of the bucker-handle meniscal tear toward the anterior of the femoral condyle was avoided during the removal of the needle, following the placement of the first implant on the capsule in all-inside repair (Figure 2). Subsequently, meniscal repair was performed through all-inside suture, 5 mm distant from the first vertical oblique suture to the posterior of the meniscus (Fast-Fix 360 Meniscal Repair System, Smith & Nephew, Andover, MA). The meniscal repair was completed with all-inside and inside-out sutures placed on the corpus of the meniscus with intervals of 5 mm between stitches, based on the tear size and stability (Figure 3). Stability of the probe was checked and the intraarticular arthroscopic fluid was drained to finalize the procedure (Video Supplement).

## Materials and methods

Twenty-three bucket-handle meniscal tears of 127 patients (18%), who underwent arthroscopic knee surgery to repair the meniscus in our institution (Baltalimani Bone Diseases Training and Research Hospital) between March 2012 and April 2015, were included in this study. Fourteen of these tears were repaired with this method. Twelve patients had medial and two lateral bucket-handle tears. All patients were operated by an orthopedic surgeon (E.Ç.) experienced in this field. No braces were used following the surgery, and knee range of motion (ROM) exercises without full-weight-bearing were given for the first six weeks. Flexion was limited to 90° during the same period. Touchdown weight-bearing was started on week two. Partial weight-bearing was allowed by week three and full-weight-bearing after week six. Full knee flexion was allowed after the eighth week. The patients were free to attend sporting activities after six months.

## Discussion

Our aim in the employment of this new technique for the repair of bucket-handle meniscal tears was to provide a simpler and safer surgical treatment for such challenging tears and a more convenient environment for suturing of these tears, especially for surgeons with limited experience.

Reduction of bucket-handle meniscal tears and maintenance of the reduction during the repair are both challenging [7]. With the introduction of new-generation, all-inside repair instruments, risks of neurovascular injury and capsular adhesion observed following the application of inside-out repairs, particularly in posterior horn tears of the meniscus, have been minimized [8]. The reasons for failure such as challenges in adjusting the tension of the sutures, leaving the implants inside the joint, and applying a second implant have been reported in utilization of these instruments and the failure of the suture occurs when the knot is not deployed in the right way or is at a wrong distance [9]. In addition, removal of the material inside the joint increases the risk of additional injuries to structures like chondral surfaces of the joint [10]. However, with the application of the first inside-out suture in our technique, a complex bucket-handle tear will transform into two simple longitudinal tears, rendering the surgery much easier and safer and preventing emergence of above complications.

A possible complication of employing the all-inside suture technique at first stage in repair of these tears is that, following the placement of the first implant behind the capsule, the needle tip may pull the torn segment toward itself when being removed and thus result in failure of the reduction. In such a situation the second implant cannot be used, rendering the suture instrument unusable and causing iatrogenic damage in the meniscus. In clinical application of this instrument, Haas et al. experienced complications five times and had to use new suture instruments [11]. Such occurrences will impose additional cost by increasing surgical and institutional expenses. However, no hand instrument or meniscal suture has failed with the application of our method and no additional or replacement supplies were required. This is a significant advantage of our technique.

Yoon et al. described an outside-in method for the reduction of bucket-handle meniscal tears and reported that the stability of the meniscus achieved is essential for the applicability of other repair methods, in addition to obtaining a proper reduction through the use of their method. Nevertheless, they also experienced challenges with the application of their technique [7]. After obtaining reduction of the meniscus with the inspection probe, the application of inside-out suture material through the posterior of the meniscus and its corpus first to maintain the reduction, and completion of the repair with all-inside sutures are the main principles of our method which can be easily applied without broad experience. Also, there are a lot of described techniques to stabilize the meniscal tear fragment temporarily by the probe or trochar. But in these techniques arthroscopy should be performed by tree portal.

The major limitation of our study was that there was no comparative result of our technique with those of other methods. Another limitation of our study was the lack of clinical outcomes. Further studies are required to demonstrate clinical comparisons or second-look arthroscopy outcomes.

Our recommendation of employment of the inside-out technique at the first stage in the repair of bucket-handle meniscal tears will allow stabilization of the meniscus, prevent loss of reduction, increase the efficacy of further all-inside sutures, and prevent unnecessary waste of suture materials. In addition, as it minimizes the irreparable damage in the meniscus, we believe this suture combination is preferable in the treatment of bucket-handle meniscal tears.

In conclusion, we believe the suture combination suggested in this study should be the choice of treatment as it is easily applicable and minimizes the risk of complications that might be encountered during repairs of meniscal tears with the all-inside technique.

## Conflict of interest

The authors declare no conflict of interest in relation with this paper.

## Supplemental Material

Application of the inside-out suture as the first suture. Then application of the second all-inside suture. After that application of the additional inside-out suture in our hybrid technique.
